# Supplementary material for: Thermodynamic modeling reveals widespread multivalent binding by RNA-binding proteins
Source: Bioinformatics. 2021 Jul 12;37(Suppl 1):i308–16. doi: 10.1093/bioinformatics/btab300 (PMC8275352; doi:10.1093/bioinformatics/btab300)
Supplement: btab300_Supplementary_Data [file btab300_supplementary_data.pdf]

Supplemental Information

Thermodynamic modeling reveals widespread multivalent binding by RNA-binding proteins

Salma Sohrabi-Jahromi,[1] Johannes Söding[1,2*]

[1] Quantitative and Computational Biology, Max Planck Institute for Biophysical Chemistry, Göttingen, Germany.

[2] Campus-Institut Data Science (CIDAS), Göttingen, Germany.

* Correspondence: soeding@mpibpc.mpg.de

Supplementary Methods

Parameter Optimization

We learn BMF model parameters by maximizing the likelihood function (equation 9 in manuscript). For an efficient optimization using stochastic gradient descent, we need to be able to compute the partial derivative of the likelihood with respect to model parameters (θ):

$$\frac{\partial LL(\Theta)}{\partial\theta} = \sum_{\mathbf{x}\in\mathcal{X}^+} \frac{1}{Z(\mathbf{x})(1-Z(\mathbf{x}))}\frac{\partial Z(\mathbf{x})}{\partial\theta} - N^+ \frac{\sum_{\mathbf{x}'\in\mathcal{X}^{\mathrm{bg}}} p_{\mathrm{bg}}(\mathbf{x}')Z(\mathbf{x}')^{-2}\times\frac{\partial Z(\mathbf{x}')}{\partial\theta}}{\sum_{\mathbf{x}'\in\mathcal{X}^{\mathrm{bg}}} p_{\mathrm{bg}}(\mathbf{x}')(1-1/Z(\mathbf{x}'))}. \tag{1}$$

We can compute the partial derivatives $\partial Z(\mathbf{x})/\partial\theta$ from the partial derivatives $\partial Z_{\mathrm{A}}(i)/\partial\theta$ and $\partial Z_{\mathrm{A}}(L)/\partial\theta$ according to equation 6 in the manuscript:

$$\frac{\partial Z(\mathbf{x})}{\partial\theta} = \frac{\partial Z_{\mathrm{B}}(x, L-1)}{\partial\theta} + \sum_{i=0}^{L-1}\frac{\partial Z_{\mathrm{A}}(x,i)}{\partial\theta}\ . \tag{2}$$

BMF parameters (θ) include binding energies of each domain to various k-mers as well as concentration parameters (S, r and p).

In the following, We define $\theta_{k,d}$ as the binding energy of domain d at k-mer k. Log-likelihood derivatives with respect to binding energies can be computed iteratively by applying the partial derivative operator on the forward algorithm of the dynamic programming (equations 1 and 2 in the manuscript):

$$\frac{\partial Z_{\mathrm{A}}(i)}{\partial\theta_{k,d}} = c_{\mathrm{AB}}e^{-E_A(i)}\left[\frac{\partial Z_{\mathrm{B}}(i-k)}{\partial\theta_{k,d}} + \sum_{j=0}^{i-k}\frac{\partial Z_{\mathrm{A}}(j)}{\partial\theta_{k,d}} - \left(Z_{\mathrm{B}}(i-k) + \sum_{j=0}^{i-k} Z_{\mathrm{A}}(j)\right)\frac{\partial E_A(i)}{\partial\theta_{k,d}}\right], \tag{3}$$

where

$$\frac{\partial E_A(i)}{\partial \theta_{k,d}} = \delta_{x(i),k}\, \delta_{d,A}\ , \tag{4}$$

and $\delta_{i,j}$ is the Kronecker delta of i and j. Similarly we can get the derivatives with respect to Z_B:

$$\frac{\partial Z_\mathrm{B}(i)}{\partial \theta_{k,d}} = \frac{\partial Z_\mathrm{B}(i-1)}{\partial \theta_{k,d}} + \sum_{j=0}^{i-k} c_\mathrm{B}(i-k-j)\, e^{-E_B(i)} \left(\frac{\partial Z_\mathrm{A}(j)}{\partial \theta_{k,d}} - Z_\mathrm{A}(j) \frac{\partial E_B(i)}{\partial \theta_{k,d}} \right)$$
$$+\, c_\mathrm{AB}\, e^{-E_B(i)} \left(\frac{\partial Z_\mathrm{B}(i-k)}{\partial \theta_{k,d}} - Z_\mathrm{B}(i-k) \frac{\partial E_B(i)}{\partial \theta_{k,d}} \right), \tag{5}$$

where

$$\frac{\partial E_B(i)}{\partial \theta_{k,d}} = \delta_{x(i),k}\, \delta_{d,B}\ . \tag{6}$$

These derivatives are computed iteratively via dynamic programming similar to Z_A and Z_B. They are initialized to zero for:

$$\frac{\partial Z_\mathrm{A}(i)}{\partial \theta_{k,d}} = 0 \text{ for all } i \in \{0, ..., k-2\} \tag{7}$$
$$\frac{\partial Z_\mathrm{B}(i)}{\partial \theta_{k,d}} = 0 \text{ for all } i \in \{0, ..., k-2\}\,. \tag{8}$$

Similarly, we can derive the partial derivative in respect to the concentration parameters (θ_c):

$$\frac{\partial Z_\mathrm{A}(i)}{\partial \theta_c} = c_\mathrm{AB}\ e^{-E_A(i)} \left(\frac{\partial Z_\mathrm{B}(i-k)}{\partial \theta_c} + \sum_{j=0}^{i-k} \frac{\partial Z_\mathrm{A}(j)}{\partial \theta_c} \right)\ , \tag{9}$$

$$\frac{\partial Z_\mathrm{B}(i)}{\partial \theta_c} = \frac{\partial Z_\mathrm{B}(i-1)}{\partial \theta_c} + \sum_{j=0}^{i-k} e^{-E_B(i)} \left(\frac{\partial Z_\mathrm{A}(j)}{\partial \theta_c} c_\mathrm{B}(i-k-j) + Z_\mathrm{A}(j) \frac{\partial c_\mathrm{B}(i-k-j)}{\partial \theta_c} \right)$$
$$+ \frac{\partial Z_\mathrm{B}(i-k)}{\partial \theta_c} c_\mathrm{AB} + Z_\mathrm{B}(i-k) \frac{\partial c_\mathrm{AB}}{\partial \theta_c}\ . \tag{10}$$

The partial derivative $\partial c_\mathrm{B} / \partial \theta_c$ with respect to concentration parameters S, r and p are (according to equation 5 in the manuscript):

$$\frac{\partial c_\mathrm{B}(d)}{\partial S} = \frac{\Gamma(d+r)}{\Gamma(d+1)\Gamma(r)} p^r (1-p)^d, \tag{11}$$

$$\frac{\partial c_\mathrm{B}(d)}{\partial p} = S \times \left(\frac{r}{p} - \frac{d}{1-p}\right) \times \exp\big(\log\Gamma(d+r)$$
$$- \log\Gamma(d+1) - \log\Gamma(r) + d\log(1-p) + r\log p\big), \tag{12}$$

$$\frac{\partial c_\mathrm{B}(d)}{\partial r} = S \times \big(\psi(d+r) + \log p - \psi(r)\big) \times \exp\big(\log\Gamma(d+r)$$
$$- \log\Gamma(d+1) - \log\Gamma(r) + d\log(1-p) + r\log p\big), \tag{13}$$

where Γ is the gamma function and ψ is its logarithmic derivative, also known as the digamma function. Note that for numerical accuracy, we have calculated the derivatives of the $\exp(\log c_{\mathrm{B}}(d))$ function, with respect to p and r.

Overall, these equations allow us to iteratively compute for any sequence $\mathbf{x}$ the partial derivatives $\partial Z_{\mathrm{A}}(i)/\partial\theta$ and $\partial Z_{\mathrm{B}}(L)/\partial\theta$ with respect to all parameters and hence derivatives of the partition function $Z(x)$ and those of the likelihood.

The thermodynamic model contains a simplification. We assume that one of the domains (A) always binds upstream of the other domain (B) and that the binding configurations A-B and B-A do not *both* contribute appreciably to the binding probability. This seems like a very plausible assumption considering that the linkers between structural domains are usually quite short, and changing the order of binding would usually result in an impossible or much less favorable (tighter) configuration of the RNA chain.

Calculation of motif entropy

To derive the entropy for each bipartite motif model, we calculate the weighted probability for each base as

$$P_b = \frac{\sum\limits_{x\in k\text{-mers}} n_{b,x}p_x + \sum\limits_{y\in k\text{-mers}} n_{b,y}p_y}{\sum\limits_{b\in N}\Big(\sum\limits_{x\in k\text{-mers}_A} n_{b,x}p_x + \sum\limits_{y\in k\text{-mers}} n_{b,y}p_y\Big)}, \tag{14}$$

where N is the set of nucleotides ($\{A, C, G, U\}$). We calculate the entropy as

$$\text{Entropy} = -\sum_{b\in N} P_b \log_2 P_b\,. \tag{15}$$

To establish a baseline for the observed entropy values, we generated artificial bipartite motifs where the k-mer probabilities are taken from the observed probabilities of an experimental set but the k-mers were shuffled. We generated 10,000 such motifs and used the resulting entropy distribution as a baseline for motif complexity.

Calculation of motif repetitiveness

To quantify the degree of sequence repetitiveness in BMF models, we calculate the highest average probability of observing a repetitive 3-mer (i.e. 'AUA', 'UUU', or 'CGC') as

$$R = \max_{a,b\in N}\left(\sqrt{p_A(aba) + p_A(bab))(p_B(aba) + p_B(bab)}\right), \tag{16}$$

where N is the set of nucleotides ($\{A, C, G, U\}$), and p_A and p_B are BMF probabilities for the first and second motif core respectively. To establish a baseline for the observed repetitiveness values, we calculated this metric for 10,000 artificial bipartite motifs, generated as described above.

BMF comparison with single-occurrence motif model

To estimate the effect of considering all binding configurations and including cooperativity in BMF, we compared its cross-validated classification performance with a spaced k-mer motif model. We created training and test sets by splitting the HTR-SELEX data with an 80 to 20 ratio. We trained BMF with core size 3 on the training data and used the learned models to predict the binding scores for each

sequence in the test set. For the spaced k-mer model, we calculated enrichment factors for each spaced k-mer in the training data and scored the test sequences by the most enriched k-mer motif. The k-mers have 6 informative positions with the pattern 1110...0111. The length of spacers (zeros in the pattern) vary between 0 and 6 and was chosen to match the best spacer length in the corresponding BMF model. To compare each model's classification power, we calculated the area under the receiver operating characteristic curve (AUROC) for all RBPs in the dataset.

Cross-platform validation of HTR-SELEX models on RNAcompete data

We trained BMF with core sizes 3, 4, and 5, as well as iDeepE, DeepCLIP, and GraphProt models on HTR-SELEX data. We then compared how these models perform at predicting bound fragments in RNAcompete datasets. RNAcompete sequences were sorted by their normalized intensities and the 2000 sequences with highest scores were assigned to the positive class, while the 2000 sequences with the lowest scores were labeled as the background set. Sequences shorter than 40 nucleotides were padded with N to generate same-length fragments. Normalized RNAcompete data was collected from http://hugheslab.ccbr.utoronto.ca/supplementary-data/RNAcompete_eukarya/.

Supplementary figures

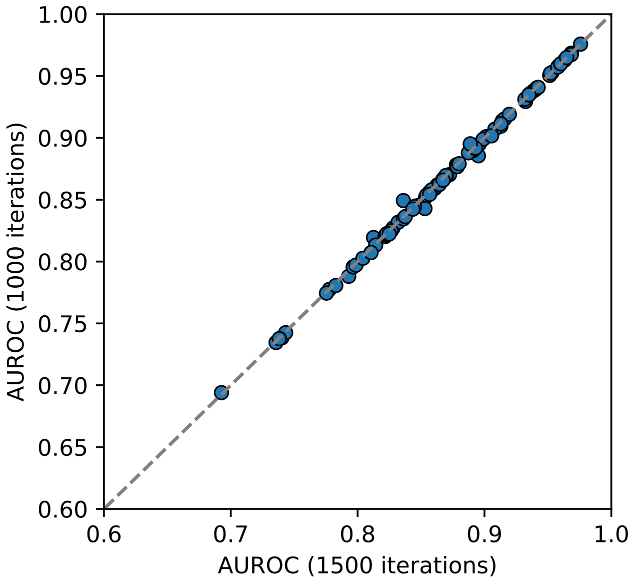

Figure S1: **BMF's predictive performance matches between 1000 and 1500 iterations of gradient descent.** AUROC values are calculated by predicting binding sites in held-out sequences of HTR-SELEX datasets (80%-20% split for training and testing). Stochastic gradient descent was performed for a fixed number of 1000 and 1500 iterations respectively.

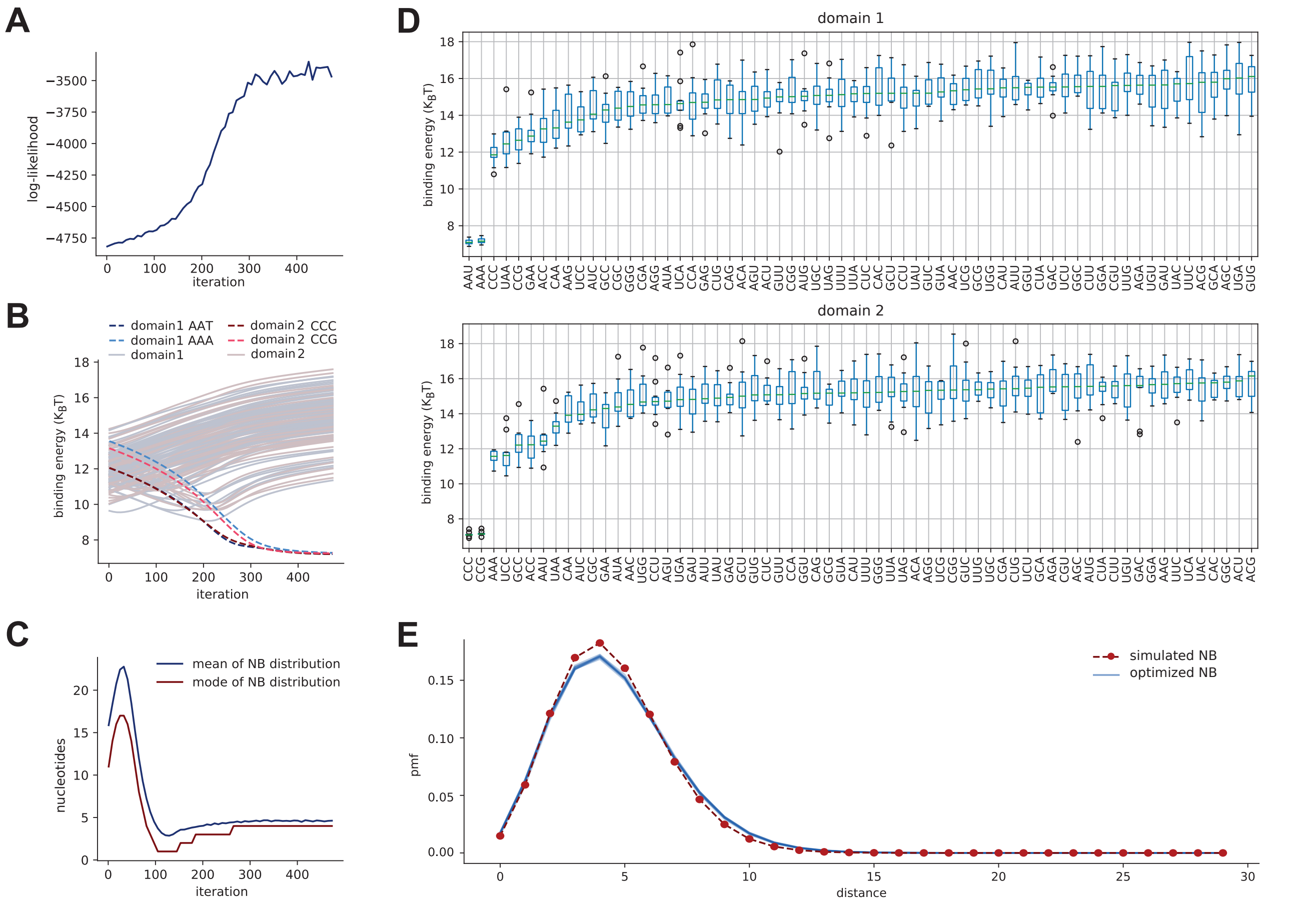

Figure S2: **BMF can reliably learn planted motifs in synthetic data.** We planted AA(A/U) followed by CC(C/G) with a distance distribution around 4 in 2000 randomly generated sequences of length 40. **(A)** Log-likelihood function increases over the iterations until reaching a plateau at the end of optimization. **(B)** The binding energies of all 3-mers are shown over BMF iterations for both binding domains. The 3-mers representing the implanted motifs are shown with brighter blue (first domain) and red (second domain)dash lines. The final values retrieved after optimization is notably lower for the highlighted 3-mers. **(C)** The mean (in blue) and mode (in red) of the NB distribution is shown over BMF's optimization iterations. The correct distance distribution is found when the LL and the energy parameters reach their plateaus. **(D)**, and **E** The distribution of final BMF parameters upon 10 random parameter initializations and subsequent optimization. Regardless of the choice of initial parameter values, BMF ends in the same optimum point in the parameter landscape.

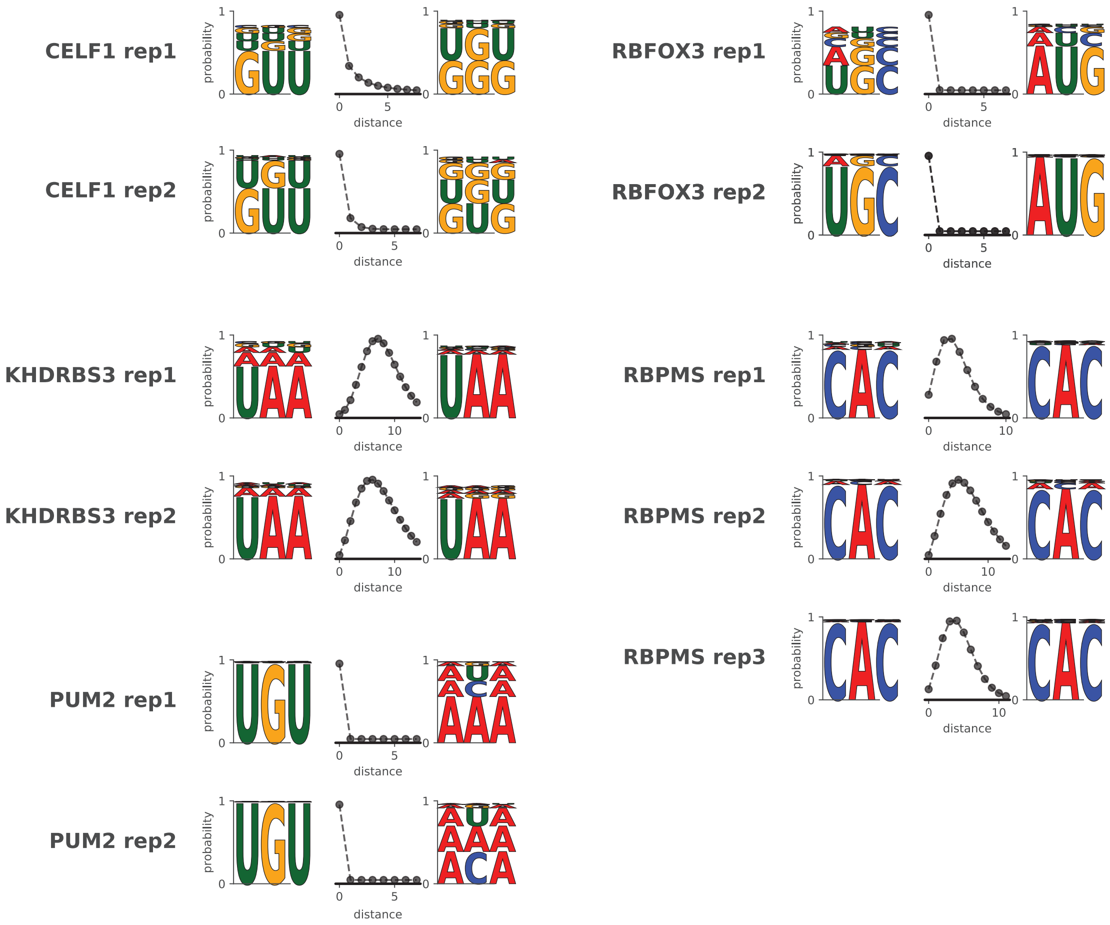

Figure S3: **Experimental HTR-SELEX replicates generate the same bipartite motif models**. Biparite binding models are shown for factors in Figure 2 for which an experimental replicate was available. The models generated for all HTR-SELEX datasets can be found in BMF GitHub repository: `https://github.com/soedinglab/bipartite_motif_finder/blob/main/data/HTRSELEX_motifs.pdf`.

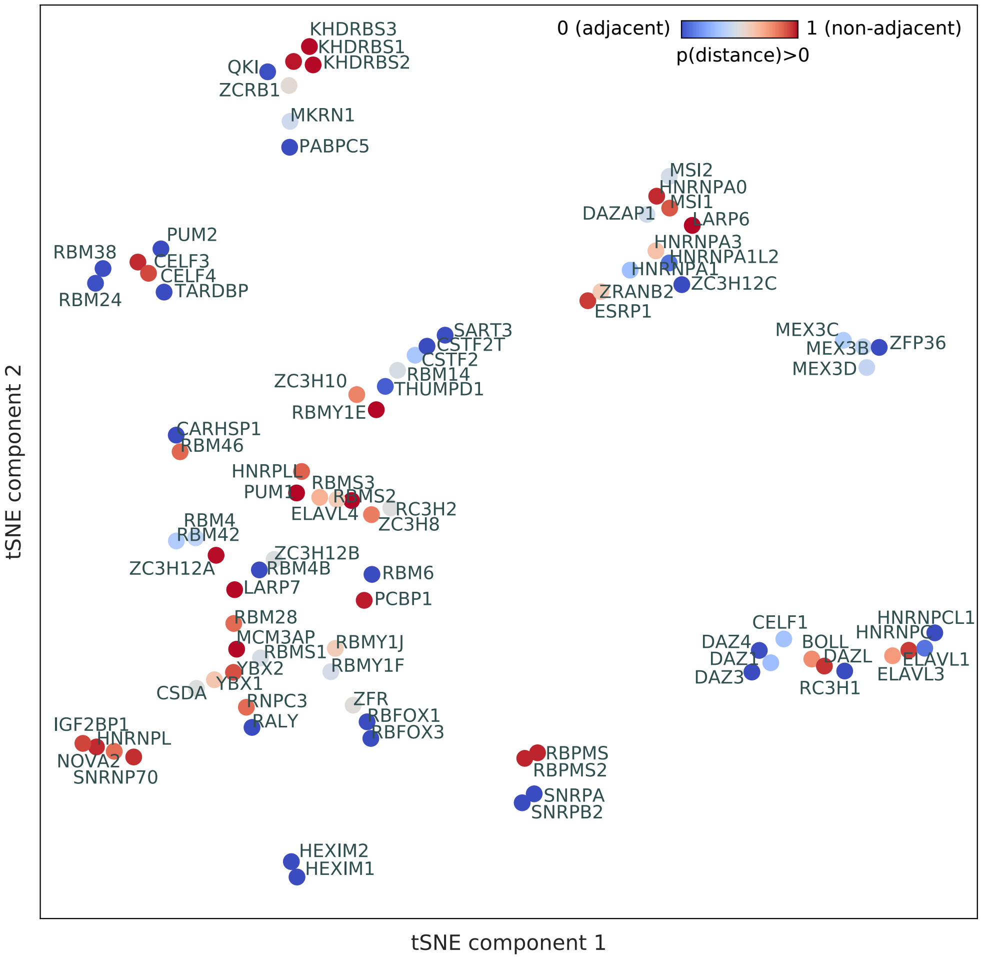

Figure S4: **RBPs in the same family have similar BMF motifs**. BMF models are clustered according to their sequence identity measured as pairwise Pearson correlation between 3-mer probabilities. Two dimensional embedding is generated via tSNE [2]. RBPs are color-coded based on the domain positioning in the NB models, as in Figure 2B, with adjacent cores colored in blue and bipartite motifs in red. Additional information on the domain composition of each RBP is provided in Table S1.

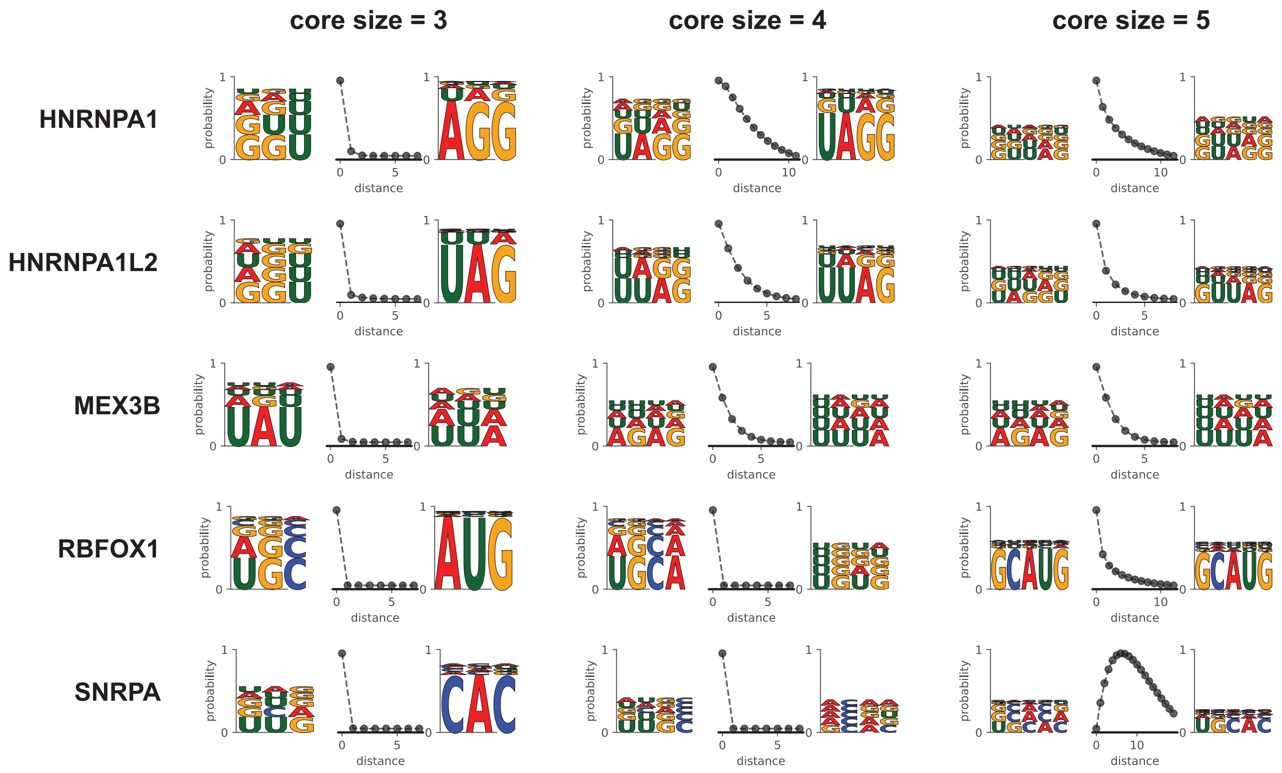

Figure S5: **Bipartite binding behaviour can arise when building longer sequence models**. Some RBPs in the HT-SELEX dataset have adjacent cores when building BMF models with 3-mers, but show bipartite binding for 4-mer and/or 5-mer models.

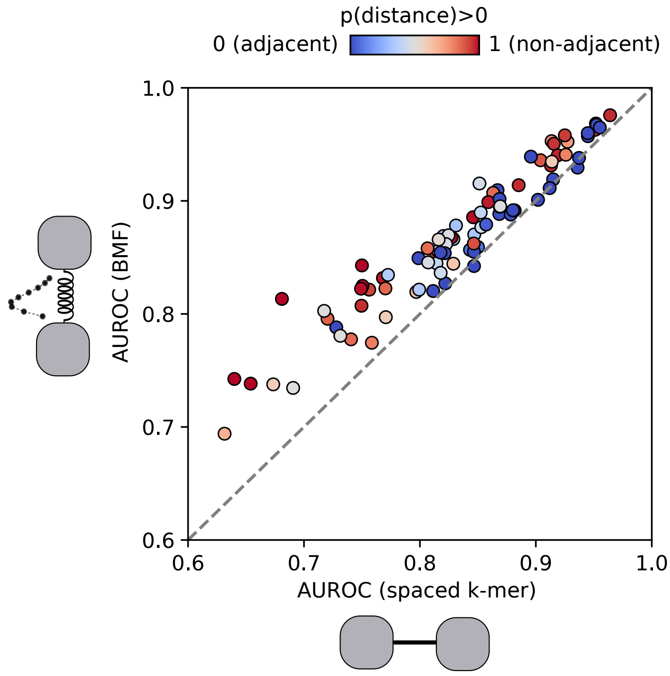

Figure S6: **Incorporating cooperativity and multivalency boosts performance of RBP binding models.** AUROC values are calculated by predicting binding sites in held-out sequences of HTR-SELEX datasets (80%-20% split for training and testing). BMF with core size 3 is compared to a single-occurence per sequence spaced *k*-mer model (see supplementary methods). RBPs are color-coded based on the domain positioning in the NB models, as in Figure 2B, with adjacent cores colored in blue and bipartite motifs in red.

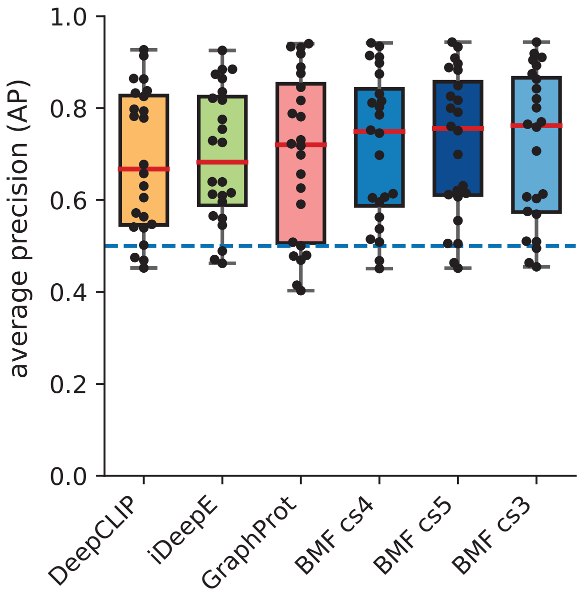

Figure S7: **Average precision (AP) scores for iDeepE, DeepCLIP, GraphProt, and BMF with motif sizes ranging from 3 to 5.** We used BMF, iDeepE, DeepCLIP, and GraphProt to identify eCLIP and PAR-CLIP RBP binding sites based on the models trained on HTR-SELEX datasets. The tools are sorted based on their median AP scores (red lines). The AP score for each RBP dataset is shown with a black dot.

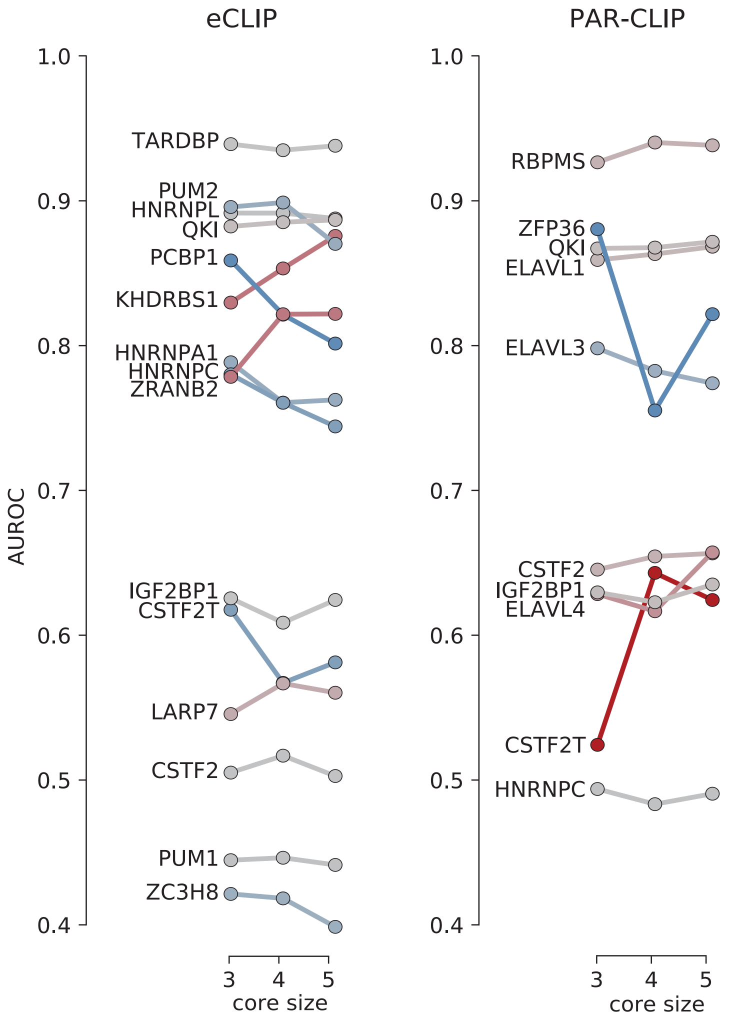

Figure S8: **Comparison of cross-platform AUROC values for BMF models with core sizes 3 to 5.** An increase in AUROC with increasing motif length is marked with red and a decrease with blue.

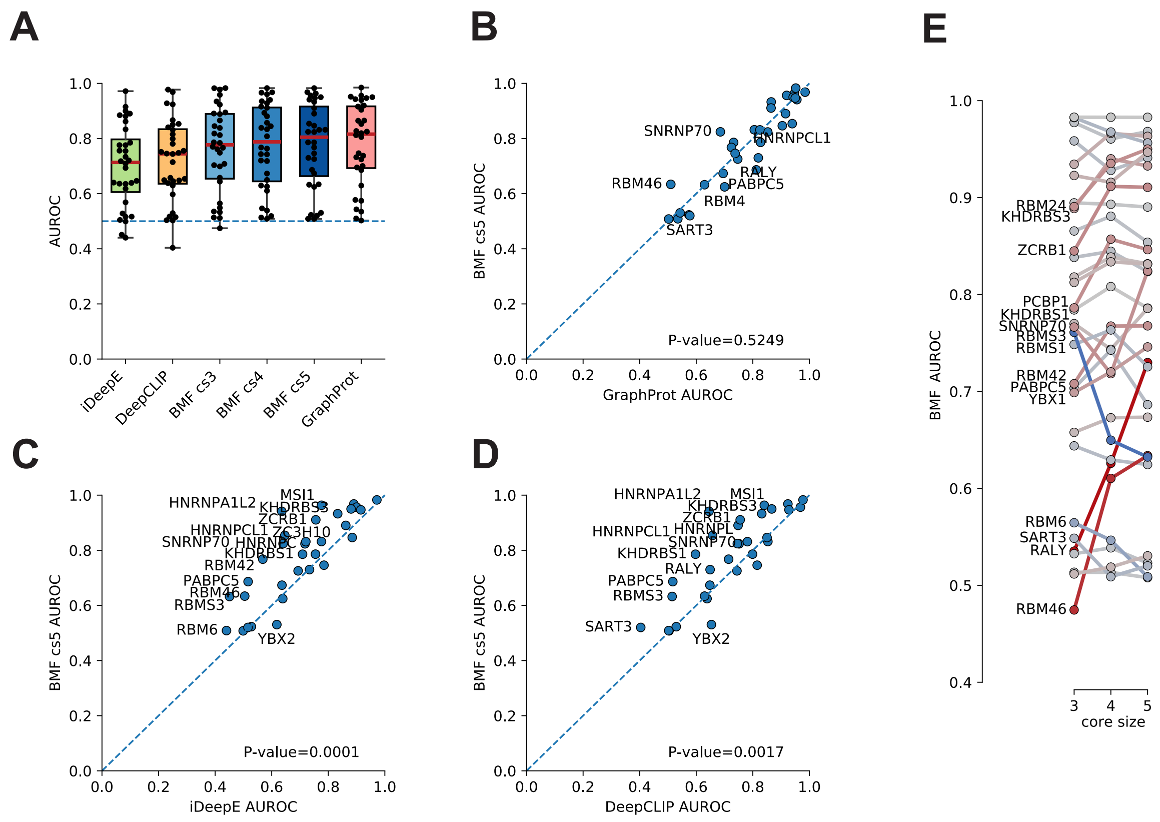

Figure S9: **Cross-platform validation of HTR-SELEX motif models used to predict binding on RNAcompete data.** We used BMF, iDeepE, DeepCLIP, and GraphProt to identify bound sequences in RNAcompete datasets after training their motif models on HTR-SELEX data. **(A)** AUROC distribution for iDeepE, DeepCLIP, GraphProt, and BMF with motif sizes ranging from 3 to 5. The tools are sorted based on their median AUROC performance. The values for each RBP dataset is shown with a black dot. **(B)** to **(D)** AUROC from BMF (core size 5) compared to GraphProt, iDeepE, and DeepCLIP. Statistical significance was assessed through Wilcoxon signed-rank tests. **(E)** Comparison of cross-platform AUROC values for BMF models with core sizes 3 to 5. In all plots AUROC values are averaged over all replicate combinations wherever replicates were available.

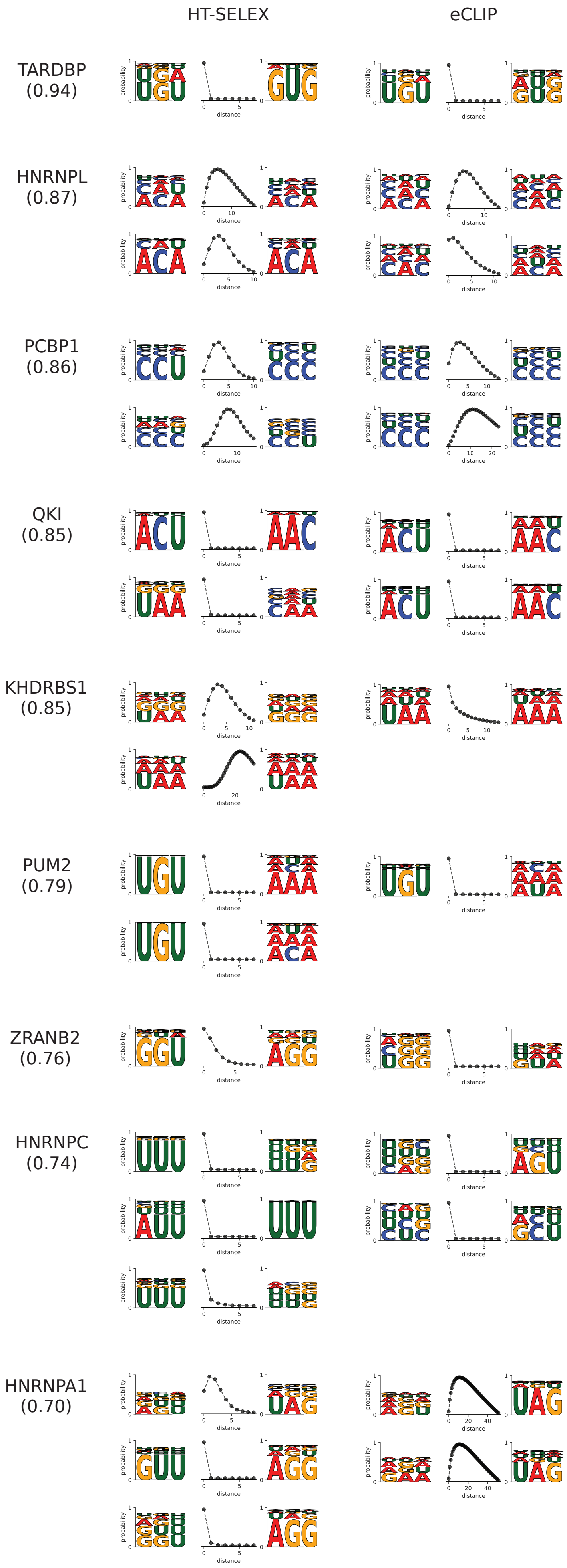
HT-SELEX
eCLIP
TARDBP
(0.94)
HNRNPL
(0.87)
PCBP1
(0.86)
QKI
(0.85)
KHDRBS1
(0.85)
PUM2
(0.79)
ZRANB2
(0.76)
HNRNPC
(0.74)
HNRNPA1
(0.70)
probability
distance

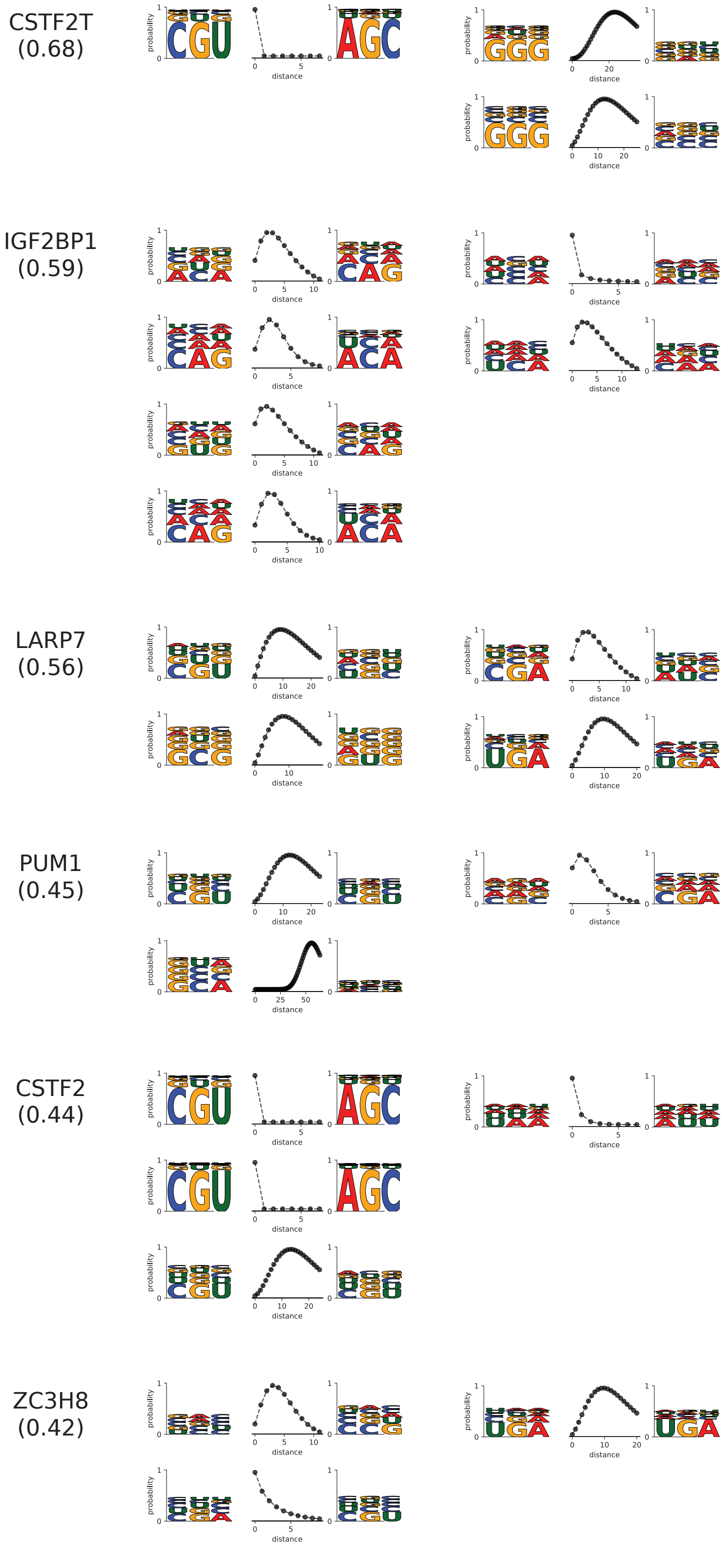

Figure S10: **Comparison of HTR-SELEX and eCLIP BMF logos**. BMF logos are sorted according to their cross-platform AUROC performance (shown in parenthesis), which is an average between BMF (with core size 3), Graphprot, and iDeepE. BMF logos were generated for all available replicates of each experimental technique.

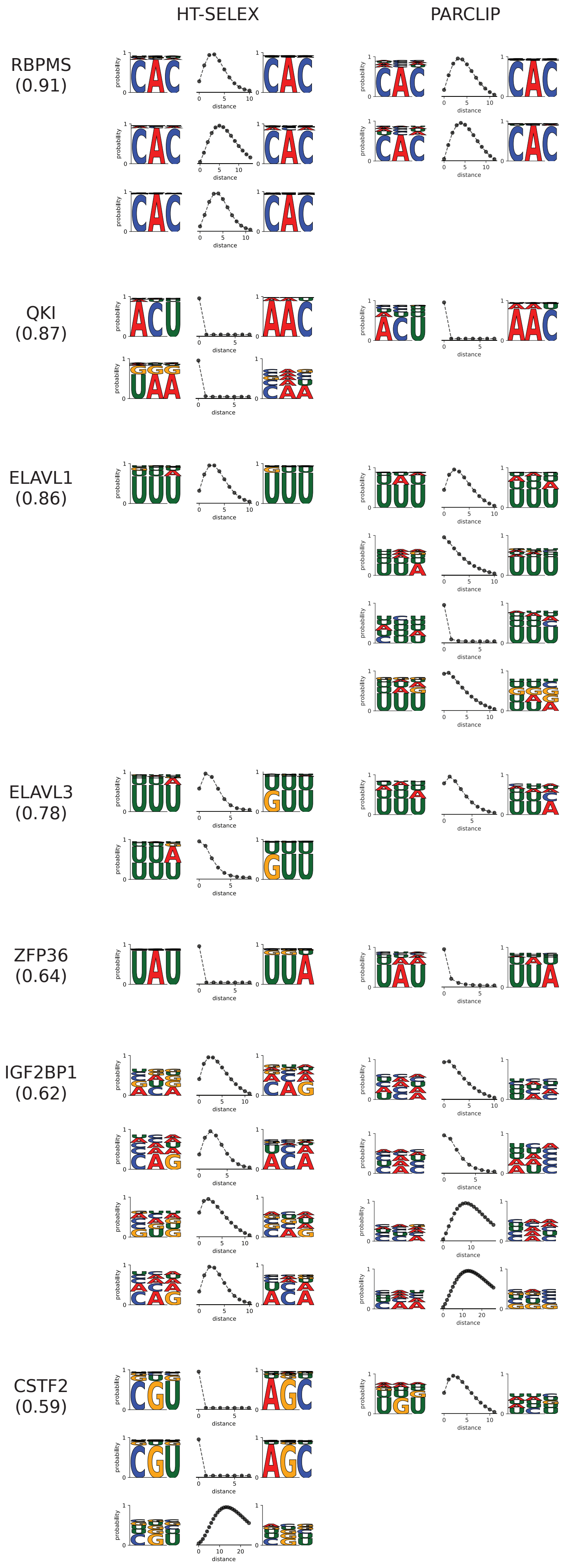
HT-SELEX
PARCLIP
RBPMS
(0.91)
QKI
(0.87)
ELAVL1
(0.86)
ELAVL3
(0.78)
ZFP36
(0.64)
IGF2BP1
(0.62)
CSTF2
(0.59)
probability
distance

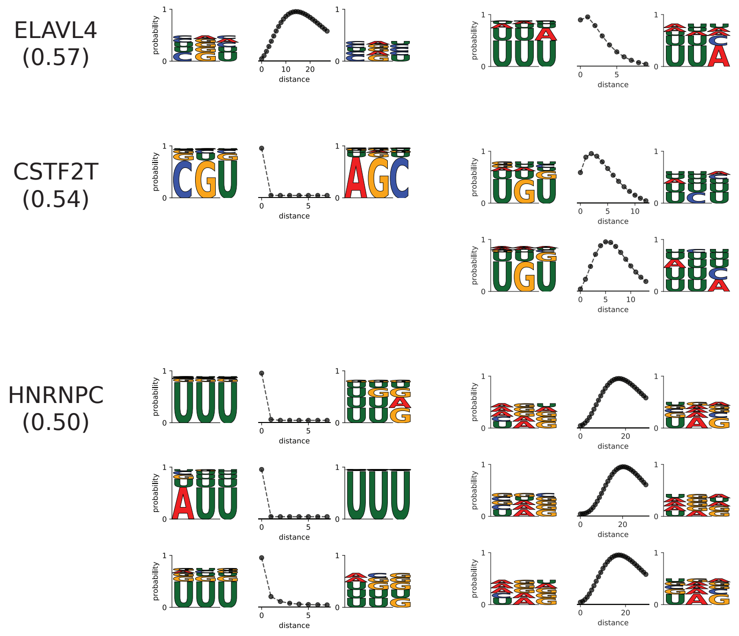

Figure S11: **Comparison of HTR-SELEX and PAR-CLIP BMF logos**. BMF logos are sorted according to their cross-platform AUROC performance (shown in parenthesis), which is an average between BMF (with core size 3), Graphprot, and iDeepE. BMF logos were generated for all available replicates of each experimental technique.

Supplementary Table

Table S1: **Domain architecture of RBP constructs in the HTR-SELEX dataset used to train BMF models**. Annotations are based on HMMER search [3] of the construct sequences used by Jolma *et al.*

RBP	CONSTRUCT ARCHITECTURE
**BOLL**	RRM_1
**CARHSP1**	CSD
**CELF1**	RRM_1, RRM_1
**CELF3**	RRM_1, RRM_1
**CELF4**	RRM_1
**CSDA**	CSD
**CSTF2**	CSTF2_hinge, RRM_1, CSTF_C
**CSTF2T**	CSTF2_hinge, RRM_1, CSTF_C
**DAZ1**	RRM_1, RRM_1, RRM_5, RRM_5
**DAZ3**	RRM_1
**DAZ4**	RRM_1
**DAZAP1**	RRM_1, RRM_1, RRM_7
**DAZL**	RRM_1
**ELAVL1**	RRM_1, RRM_1, RRM_1, RRM_5, RRM_5
**ELAVL3**	RRM_1, RRM_1, RRM_1, RRM_5, RRM_5
**ELAVL4**	RRM_1, RRM_1, RRM_1, RRM_5, RRM_5
**ESRP1**	RRM_1
**HEXIM1**	HEXIM
**HEXIM2**	HEXIM
**HNRNPA0**	RRM_1, RRM_1
**HNRNPA1**	RRM_1, RRM_1, HnRNPA1
**HNRNPA1L2**	RRM_1, RRM_1, HnRNPA1
**HNRNPA3**	RRM_1, RRM_1
**HNRNPC**	RRM_1
**HNRNPCL1**	RRM_1
**HNRNPL**	RRM_5, RRM_5, RRM_1, RRM_1, RRM_8
**HNRPLL**	RRM_5, RRM_5, RRM_1, RRM_1, RRM_8
**IGF2BP1**	KH_1, KH_1, KH_1
**KHDRBS1**	KH_1
**KHDRBS2**	Qua1, Sam68-YY, KH_1
**KHDRBS3**	Qua1, Sam68-YY, KH_1
**LARP6**	La, SUZ-C
**LARP7**	RRM_3
**MEX3B**	KH_1, KH_1
**MEX3C**	KH_1, KH_1
**MEX3D**	KH_1, KH_1
**MKRN1**	zf-CCCH_4, zf-CCCH_4, zf-CCCH_4, zf-CCCH, zf-CCCH, zf-CCCH, zf_CCCH_4, zf-C3HC4, zf-RING_UBOX
**MSI1**	RRM_1, RRM_1
**MSI2**	RRM_1, RRM_1
**NOVA2**	KH_1, KH_1
**PABPC5**	RRM_1, RRM_1, RRM_1, RRM_1, RRM_5, RRM_5
**PCBP1**	KH_1, KH_1, KH_1
**PUM1**	PUF, PUF, PUF, PUF, PUF, PUF, PUF, PUF
**PUM2**	PUF, PUF, PUF, PUF, PUF, PUF, PUF, PUF
**QKI**	STAR_dimer, Quaking_NLS, KH_1
**RALY**	RRM_1
**RBFOX1**	RRM_1, Fox-1_C
**RBFOX3**	Fox-1_C, RRM_1
**RBM14**	RRM_1, RRM_1, RRM_5, RRM_5
**RBM24**	RRM_1
**RBM28**	RRM_1, RRM_1, RRM_1
**RBM38**	RRM_1
**RBM4**	RRM_1, RRM_1, RRM_5, RRM_5, zf-CCHC
**RBM42**	RRM_1
**RBM46**	RRM_1, RRM_1, RRM_1, DND1_DSRM
**RBM4B**	RRM_1, RRM_1, RRM_5, RRM_5, zf-CCHC
**RBM6**	OCRE, G-patch
**RBMS1**	RRM_1, RRM_1
**RBMS2**	RRM_1, RRM_1
**RBMS3**	RRM_1
**RBMY1E**	RRM_1
**RBMY1F**	RBM1CTR, RRM_1
**RBMY1J**	RBM1CTR, RRM_1
**RBPMS**	RRM_1
**RBPMS2**	RRM_1
**RC3H1**	ROQ_II, zf-RING_UBOX, zf-CCCH, zf-C3HC4
**RC3H2**	ROQ_II, zf-CCCH, zf-RING_UBOX
**RNPC3**	RRM_1
**SART3**	RRM_1, RRM_1
**SNRNP70**	U1snRNP70_N, RRM_1
**SNRPA**	RRM_1, RRM_1, RRM_5, RRM_5
**SNRPB2**	RRM_1, RRM_1, RRM_5, RRM_5
**TARDBP**	TDP43_N, RRM_1, RRM_1
**THUMPD1**	THUMP
**YBX1**	CSD
**YBX2**	CSD
**ZC3H10**	zf-CCCH, zf-CCCH, zf-CCCH_2, zf-CCCH_2, zf-CCCH_2
**ZC3H12A**	RNase_Zc3h12a, Regnase_1_C, UBA_6
**ZC3H12B**	RNase_Zc3h12a
**ZC3H12C**	RNase_Zc3h12a
**ZC3H8**	zf_CCCH_4, zf-CCCH, zf-CCCH, zf-CCCH_4, zf-CCCH_2, zf-CCCH_2, zf-CCCH_2
**ZCRB1**	RRM_1, RRM_5, zf-CCHC
**ZFP36**	zf-CCCH, zf-CCCH
**ZFR**	zf-met, zf-C2H2_jaz
**ZRANB2**	zf-RanBP, zf-RanBP

References

1 Jolma, A., Zhang, J., Mondragón, E., Morgunova, E., Kivioja, T., Laverty, K. U., Yin, Y., Zhu, F., Bourenkov, G., Morris, Q., *et al.* (2020). Binding specificities of human RNA-binding proteins toward structured and linear RNA sequences. *Genome Res*, **30**(7), 962–973.

2 Maaten, L. v. d. and Hinton, G. (2008). Visualizing data using t-SNE. *Journal of machine learning research*, **9**(Nov), 2579–2605.

3 Potter, S. C., Luciani, A., Eddy, S. R., Park, Y., Lopez, R., and Finn, R. D. (2018). HMMER web server: 2018 update. *Nucleic Acids Research*, **46**(W1), W200–W204.
